# Analysis of the Definition and Utility of Personal Health Records Using Q Methodology

**DOI:** 10.2196/jmir.1781

**Published:** 2011-11-29

**Authors:** Jeongeun Kim, David W Bates

**Affiliations:** ^1^College of NursingSeoul National UniversitySeoulRepublic Of Korea; ^2^Research Institute of Nursing ScienceSeoul National UniversitySeoulRepublic Of Korea; ^3^Interdisciplinary Program of Medical InformaticsSeoul National UniversitySeoulRepublic Of Korea; ^4^Division of General Internal MedicineBrigham and Women's HospitalBoston, MAUnited States; ^5^Harvard Medical SchoolBoston, MAUnited States; ^6^Partners Information SystemsPartners HealthCareBoston, MAUnited States

**Keywords:** Personal health record, P-sample, Q-sample, Q-statement, qualitative research, self-efficacy

## Abstract

**Background:**

Personal health records (PHRs) remain a relatively new technology and concept in practice even though they have been discussed in the literature for more than 50 years. There is no consensus on the definition of a PHR or PHR system even within the professional societies of health information technology.

**Objective:**

Our objective was to analyze and classify the opinions of health information professionals regarding the definitions of the PHR.

**Method:**

Q methodology was used to explore the concept of the PHR. A total of 50 Q-statements were selected and rated by 45 P-samples consisting of health information professionals. We analyzed the resulting data by using Q methodology-specific software and SPSS.

**Result:**

We selected five types of health information professionals’ opinions: type I, public interest centered; type II, health information standardization centered; type III, health consumer centered; type IV, health information security centered; and type V, health consumer convenience centered. The Q-statements with the highest levels of agreement were as follows: (1) the PHR is the lifetime record of personal health information, (2) the PHR is the representation of health 2.0, and (3) security is the most important requirement of the PHR. The most disagreed-with Q-statements were (1) the PHR is a paper-based system, and (2) it is most effective to carry the PHR information in USB storage.

**Conclusion:**

Health information professionals agree that PHRs should be lifetime records, that they will be useful as more information is stored electronically, and that data security is paramount. To maximize the benefits of PHR, activation strategies should be developed and extended across disciplines and professionals so that patients begin to receive the benefits associate with using PHRs.

## Introduction

Recently, personal health records (PHRs) have been receiving increased attention from both the information and communications technology industry and academia as tools for consolidating, recording, and self-managing personal health information, as well as enabling self-efficacy, or the ability of patients to manage their own health. In the United States, PHRs such as Epic’s MyChart, Dossia, and Microsoft’s HealthVault that allow users to manage all of their health information in a single application are gaining many satisfied users, and many health care organizations have also built PHRs internally. According to the California Health Association’s research, 1 out of 14 Americans use PHRs, and the number has doubled in the past 2 years. Also, a majority of the users are of high-income demographic status, and they tend to be younger than those who do not use PHRs. However, low-income and older patients are also increasingly adopting PHRs, as are those with chronic illnesses [1]. This may be because the system provides benefits for health management regardless of income or age. In one survey, two out of three responders expressed concerns about the security and privacy of their health information, but responders’ concerns were reduced after experiencing the many benefits of PHRs. Additionally, it is predicted that the quality of life may even increase and the cost of health care will fall as PHRs become more widely used [2]. The primary participants in the PHR industry can be divided into service providers (such as the medical, health management, and information technology industries), consumers who use PHR services, and central and local governments that support the development and advance of the technology. To realize the goals of PHRs, the perspectives of all these participants need to be considered and fairly reflected [3].

The Healthcare Information Management and Systems Society has defined PHRs as follows [4]:

An electronic Personal Health Record (“PHR”) is a universally accessible, layperson comprehensible, lifelong tool for managing relevant health information, promoting health maintenance and assisting with chronic disease management via an interactive, common data set of electronic health information and e-health tools. The ePHR is owned, managed, and shared by the individual or his or her legal proxy(s) and must be secure to protect the privacy and confidentiality of the health information it contains. It is not a legal record unless so defined and is subject to various legal limitations.

The Medical Library Association/National Library of Medicine Joint Electronic Personal Health Record Task Force also examined the state of PHRs in an extensive review in 2010. After examining various existing definitions, they provide the following working definition [5]:

Electronic personal health record (PHR): a private, secure application through which an individual may access, manage, and share his or her health information. The PHR can include information that is entered by the consumer and/or data from other sources such as pharmacies, labs, and health care providers. The PHR may or may not include information from the electronic health record (EHR) that is maintained by the health care provider and is not synonymous with the EHR. PHR sponsors include vendors who may or may not charge a fee, health care organizations such as hospitals, health insurance companies, or employers.

We recently published a systematic review on the history and trends of PHR research [6]. To assess the research efforts concerning the PHR to date, we searched the literature on research involving PHRs and have summarized the results, as well as describing how the topics assessed have evolved over time. For the search strategy, we queried PubMed, which returned 695 results. Through one-by-one analysis, we removed the results with the acronym PHR but with different definitions. In the end, we analyzed a total of 229 articles. The first appearance of PHR in an academic journal was in Germany in 1969—“Personal record linkage,” in *Methods of Information in Medicine Supplement*. However, forms were, of course, not computerized at that time, so the early literature on the PHR refers to a simple collection of paper. In other words, the PHR in a historical context was a simple collection of notes containing information on one’s health, and early studies of PHRs focused on such paper records. The shift to patient-centeredness was found afterward, and the “P” for personal in PHR was frequently used as an acronym for patient in the 1990s. Also, the phrase *personally controlled health records* strongly expresses the rights of control over one’s personal records. A similar but not identical example of the use of P as an acronym for parent was published in 1993, in the phrase *parent-held record*. The PHR started to be accepted as a separate concept from the electronic medical record (EMR) with the use of phrases such as *personal medical record* (1995) and *computer-generated patient-held medical record* (1996). The distinction between digitized and paper records in the medical field began when computerized records became the standard, and the word *electronic* was added to PHR in order to distinguish it from past paper records. In the middle of the 21st century, as the discussion of electronic health records (EHRs) became increasingly common, the term *personal* was added to EHR. This is also the period when the phrases *personal health application*, *personal health information*, *personal health folder*, and *personal health record books* came into use. As privacy and security were stressed, PHR sometimes referred to *protected health records*.

Even though the beginning of PHR research goes as far back as the 1960s, it was followed by a period of little endeavor. In the 1960s, several studies of PHRs per year were published, and this trend remained consistent until the early 2000s, when the number rapidly increased. This trend is the result of the emergence of the patient-centered care paradigm and the acknowledgment of the PHR as an important means of patient safety and eHealth because the electronic PHR can be accessed digitally from anywhere and at anytime. Additionally, the advance of Internet and information technology has enabled various enhancements of PHR functionality and expansion of applications.

In 229 articles, we analyzed the research participants, methods, and target diseases of 172 articles with abstracts in this previously published study [6]. The effects of the PHR on disease and health management were the most frequent research topics, followed by the required features of the PHR. Additionally, some studies dealt with application analysis in public health, which was initially deemed a crucial function of the PHR. As the history of the PHR is relatively short, several articles addressed the predictions regarding the future direction and the implications of PHRs. Naturally, the PHR literature overlaps at times with that around EHRs and EMRs, and few articles have made an effort to clearly delineate their differences. Given the nature of PHRs, privacy and security issues are included frequently.

The most frequently used method for PHR research was the survey method. The second most frequently used method was to analyze and test the PHR, where the focus of the studies was to investigate the various perspectives of PHR users through interviews and focus groups. In terms of the PHR being a newly developed record of health management, there were studies on recommending the initial developmental directions. A large portion fell under the *o* *ther* category because there exists a large number of varied approaches in studying PHR, which reflects the absence of a unified approach.

As such, the understanding of PHRs may differ depending on the unique perspective of each academic institution, industry, and related field. Thus, there may still be a lack of consensus in understanding what a PHR is, both conceptually and as it can be practically instantiated. This suggests that the meaning of the PHR might benefit from study, with special focus on the expert opinions from those who are actively researching and developing PHRs. An accurate understanding of the perspective of PHR experts may be valuable in considering the developmental directions and potential utility of PHRs.

In studying a new concept with an incomplete definition, such as the PHR, it is important to conduct investigative research, but it is also necessary to try to describe the subject phenomenon from a unique perspective. William Stephenson suggested Q methodology as a means of dialectically compositing the tradition of opposition methodologies such as quantitative and qualitative research, objectivity and subjectivity research, explanation and understanding methods, naturalism and humanism, and positivism and antipositivism [7]. PHRs are at a stage of development and consensus establishment. This makes PHRs a suitable application for Q methodology, as its primary objectives are exploring new and unfamiliar phenomena and those that require further development. Through categorical analysis of PHR experts’ opinions using this method, this research considers the future understanding of PHRs, as well as its current utility and further developmental directions.

## Methods

### Step 1: Selection of the Q-Sample

Q methodology is a research method used to study people’s “subjectivity”—that is, their viewpoints [8]. To study participants’ subjectivity, Q methodology uses self-referencing statements (Q-samples), which refer to phrases that project the responders’ emotions or expectations instead of facts. A group of such phrases is referred to as a Q-population, and it is obtained through literature surveys and interviews regarding the research protocol. Hundreds of Q-populations are sampled by means of a literature survey and interviews, and Q-samples are selected by random and systematic sampling methods. In its first stage, this study sampled Q-populations regarding the PHR. Initially, broad literature surveys were used to collect diverse definitions and descriptions of PHRs, followed by consolidation of similar meanings and expressions. Excluding slight differences in expressions and word arrangements, the number of specific arrangements of words in definitions of PHRs available in the published literature is finite. This signifies mostly common opinions among scholars and experts regarding the major concepts. Among the available PHR Q-samples, we selected 50 Q-statements, which we divided into 5 categories: (1) characteristics, (2) functionality, (3) form, (4) requirements, and (5) business model. We selected these statements to ensure accuracy, maximize comprehensiveness, and include a variety of accurate positive, negative, and neutral statements. As a result, the Q-statements used herein consisted of 13 characteristic statements, 11 functionality statements, 11 form statements, 7 requirement statements, and 8 business model statements.

### Step 2: Selection of the Person-Sample

Because Q methodology deals with differences in individual perspectives on relative importance, and not differences between individuals themselves, the number of person-samples (P-samples) included is not restricted. Rather, our research protocol is based on the small-sample doctrine [9]. Thompson [10] stated that opinions are best assessed through the following 5 groups: (1) those with special interest, (2) those who can judge and provide dispassionate interest, (3) those with authorities and expertise, (4) those with general interest but no special expertise, and, finally, (5) those who are uninformed and/or uninterested. The current stage of the research is not focused on all PHR users, but on the following 3 categories among the Thompson schemata who represented the P-samples: (1) special interest: PHR development executives and staff, research staff of PHR development projects; (2) authorities and experts: medical and health informatics professors, doctors, and nurses; and (3) class interests: medical and health informatics graduate students. We asked the Korean Society of Medical Informatics, which is the representative and the largest group for this professional discipline, to recommend experts for this domain, and we then invited those experts to participate voluntarily. Snowball sampling and personal contacts through professional networking were also used to reinforce the invitation of experts to form the valid P-samples. The institutional review board of the principal author’s university reviewed and approved the research, and informed consent was collected from the participants.

### Step 3: Q-Sorting

The Q-sorting stage of Q methodology requires researchers to arrange the P-sample statements into distributions according to individual degrees of agreement. In the present study, this consisted of arranging the 50 Q-sample statements into priority groups with limits on how many statements can belong to each group, starting from the highest degree of agreement to the lowest. Statistically normal distribution was used as a forced distribution to specify the limits on the priority groups (Table 1). Q-samples in Q methodology represent a portion of subjective human opinion, and thus require a systematic forced distribution of the relative importance of statements instead of using an individual scoring system. The research was conducted from June 14 to 30, 2010, and the data were collected by individual interviews with P-samples, which included explanations of the objectives and methods of the research. In Q methodology, the participants are asked to provide further descriptions of the 2 statements with the largest opposing degree of agreement to aid further in Q-factor interpretation. This was included in the explanation during the interview, and investigative analysis was conducted accordingly.

**Table 1:** Distribution of Q-sorting

	Disagree			Neutral			Agree		
Score	–4	–3	–2	–1	0	1	2	3	4
Number of Q-samples^a^	2	4	6	8	10	8	6	4	2

^a^ N (total number of Q-samples) = 50.

### Statistical Analysis

**Figure 1:**
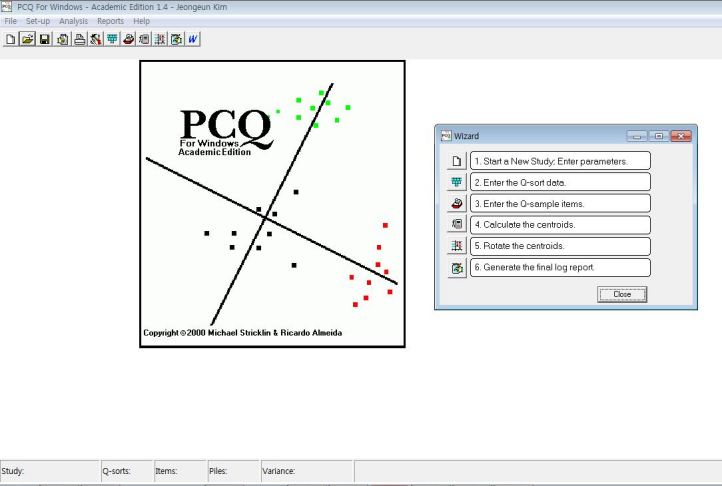
PCQ for Windows.

**Figure 2:**
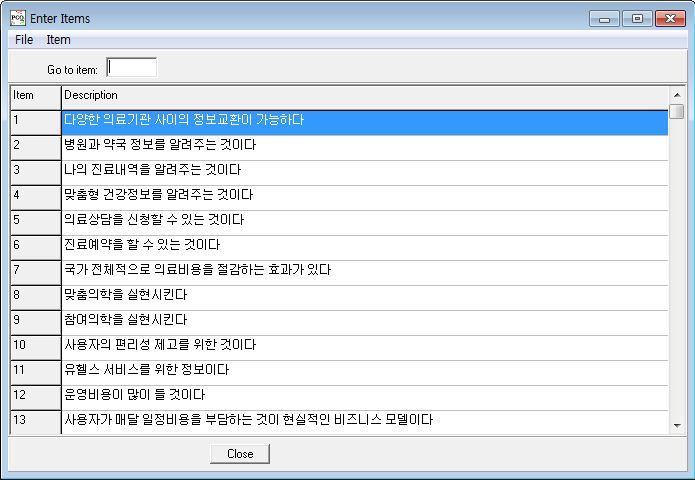
Sample data entry layout for Q-sample items (English translation of items in Multimedia Appendix 1) in PCQ for Windows.

**Figure 3:**
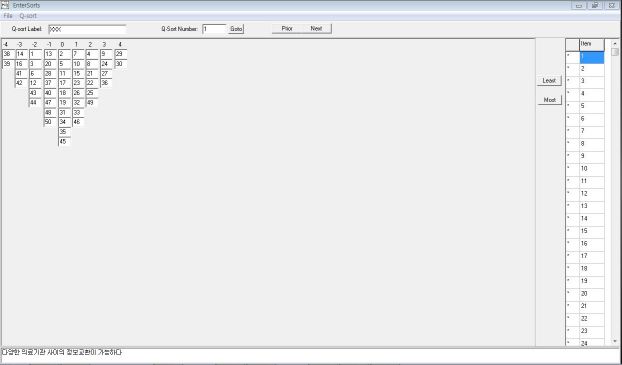
Sample data entry layout for the Q-sort data in PCQ for Windows.

Q methodology analysis is conducted through special software packages such as PCQ for Windows (PCQ Software, Portland, OR, USA), which we used in the present research (Figure 1). Figure 2 and Figure 3 show representative data layouts. In addition, we used SPSS version 19.0 (IBM Corporation, Somers, NY, USA) to reinforce the data interpretation.

## Results

### General Characteristics of Participants

We chose 45 participants for the study: 29 male and 16 female. Fields of expertise included 22 medical experts (9 doctors and 13 nurses) and 23 nonmedical experts (informatics engineering, computer engineering, genetics engineering, library and information science, etc). The average age of participants was 36.9 years (12 in their 20s, 16 in their 30s, 12 in their 40s, and 5 in their 50s).

### Categorization of Experts’ Opinions Regarding PHR According to Importance

Data were collected and analyzed using factor analysis in PCQ for Windows and SPSS 19.0. We used 45 Q-sorts as variables. The correlation coefficient was calculated and the correlation matrix was analyzed. In factor extraction, a larger-than-expected number of 18 factors had eigenvalues higher than 1 and, as it was impossible to apply these factors to factor rotation, extraction was based on the number of factors that was meaningful. The standards for selecting the number of factors were determined by inspecting the scree plot, which graphs the eigenvalue against the factor number for areas of sudden decrease in eigenvalues. Additionally, to determine the number logically, we compared and analyzed the results from setting the factor number to 5, 6, or 10, and we finally determined that the appropriate number was 5. The method used for factor extraction was principle component analysis, and the method of rotation was based on the results of processing through varimax rotation with Kaiser normalization repeated seven times. The resulting total variance of the 5 factors was 41.4%. On the basis of these analyses, we categorized expert opinions regarding PHR into 5 types (Table 2). The concepts that make up these 5 causes were organized and interpreted based on abduction—deriving a hypothesis from the observed facts—and descriptive processes.

**Table 2:** Personal health record statements with the strongest agreement and disagreement

Item number		Statement	Occurrences
**Strongest agreement statements (+5)**			
	21	It is a lifetime health record of an individual	15
	29	It is a realization of health 2.0 with the participation of clinical consumers	7
	36	Its security is of utmost importance	7
	33	It requires the protection of privacy regulation	6
	34	It needs to be accessible anytime, anywhere	6
	35	Its standardization is crucial	6
	1	It is possible to exchange data among a variety of medical institutions	4
**Strongest disagreement statements(–5)**			
	44	It is paper based	29
	50	It is most effective to store it in portable USB memory sticks	8
	24	It is not a legal document	6
	39	It requires accreditation by the government	6

#### Type I: Public Interest Centered

The eigenvalue of type I was 6.5 and the variance percentage was 14.6%; 15 participants belonged to this group. This group considered the PHR to be a lifetime health record of an individual, that it requires the protection of privacy regulation, and that security is of the utmost importance. Paper-based and USB stick-based portability received low ratings, and whether the document is a legal document was questioned. Additionally, this group perceived the business model, in which the users pay a monthly fee, as impractical. In other words, this group regarded legislation and security as of the highest priority, and objected to models in which a significant burden was placed on individual users.

#### Type II: Health Information Standardization Centered

The eigenvalue of type II was 3.4 and the variance percentage was 7.5%; 7 participants belonged to this group. The group also agreed that the PHR should be a lifetime health record of an individual, and considered the standardization of the PHR crucial. This group also viewed the idea of paper- or USB stick-based portable PHRs unfavorably, in addition to having negative opinions regarding data exchange among a variety of medical institutions. In other words, they considered the one main functionality of the PHR to be information exchange, and because this is currently not realized, standardization needs to be an early focus of development.

#### Type III: Health Consumer Centered

The eigenvalue of type III was 2.9 and the variance percentage was 6.6%; 9 participants belonged to this group. This group considered the PHR to be a realization of health 2.0 with the participation of clinical consumers, and strongly sided with consumerism. This group did not consider the completeness of information, nor accreditation by the government, to be a high priority. Similar to the other groups, they did not favor the paper-based PHR, nor a business model based on advertisement revenue.

#### Type IV: Health Information Security Centered

The eigenvalue of type IV was 2.9 and the variance percentage of 6.5%; 7 participants belonged to this group. The group considered the PHR to be a lifetime health record of an individual that requires stringent security, but disagreed that it is not a legal document and should not be paper based. This concept of the PHR shows a similarity with type I, but with a lower rank.

#### Type V: Health Consumer Convenience Centered

The eigenvalue of type V was 2.7 with a variance percentage of 6.0%; 7 participants were included in this group. This group considered the PHR to be a realization of health 2.0 with the participation of clinical consumers, and they felt strongly that the PHR needs to be accessible anytime, anywhere. However, this group was similar to type IV, in that the group questioned whether accreditation by the government was crucial, and questioned the effectiveness of paper- or USB stick-based portability.

### Consensus Regarding the Important Characteristics of PHR

The mixed research method of qualitative analysis along with quantitative methods, such as Q methodology, does not place great importance on the frequency, but rather on the weight, of meaning or relative relationships of the subject phenomena. In other words, a statement by itself has a meaning, but it also gains another meaning when relative comparison is made with other statements [11]. Therefore, the focus is not on how frequently the statement has been agreed upon, but rather on consolidating the statements that participants can commonly agree upon. Ultimately, current expert opinion of the PHR possesses the primary characteristics presented in Table 2.

## Discussion

We found that there was broad agreement that the PHR should be a lifetime health record of an individual, and it primarily requires the participation of clinical consumers. Respondents felt that other approaches, such as placing the PHR on a USB, and requiring consumers to pay a monthly fee were less likely to be practical. As expected, the different groups had differing perspectives regarding which aspects of the PHR need attention most urgently, with the largest group focusing on public interests and smaller groups focusing on a health information-centered approach, a consumer-centered approach, security as the central concern, and consumer convenience as the primary issue.

The Q methodology used in this research highlights specific behaviors in a group or quantifies the minority groups, thereby reflecting the general behavior of a larger group by studying a fraction of the group. We hypothesized that many aspects of the PHR would be divided into diverse groups and aimed to extract the primary concepts. According to Brown, the Q-sort of 50 statements applied to 45 participants and in cause analysis with a characteristic coefficient larger than 1 are both sufficient for drawing a conclusion [9]. On analysis, the topic of discussion is the categorization of opinion types among the experts. This means that these types must be considered primary concepts with regard to user uptake and use in future research on and industrialization of PHR. Because of the nature of Q methodology, this research does not assert that the result is statistically proven, which would require additional research. The 5 types extracted in this research are not statistically confirmed but are identified as impressionistic conclusions. Other research employing Q methodology discusses similar points [12]. The types of expert opinions regarding PHR identified in this study require further supplementation and proof through research efforts with a separate methodology.

### Conclusions

The PHR, which is appropriately receiving close attention from the medical and information technology industries, is likely to be widely adopted soon by large numbers of clinical consumers in developed countries. For the PHR to be efficiently used by the general public, an initial understanding of future developers’ and users’ opinions and preferences is required. Simultaneously, an accurate understanding and categorical analysis of opinions of those experts who lead the development and growth of PHR will be valuable to its adoption and expansion. In this research, we used Q methodology to categorize expert opinions on PHR. We identified 5 categories of perspectives centered on public interest, health information standardization, the health consumer, health information security, and health consumer convenience. Clearly, these are all important domains of the PHR that deserve attention. The medical industry should be developing detailed strategies for product development that address all these dimensions in order to win the support of people from all 5 perspectives. If PHRs are to achieve their considerable potential for improving health, they will need to contain sufficient content to be attractive to consumers, address their main concerns about areas such as security, and at the same time be based on business models that are successful in the long term. The domains that we identified are all going to continue to be important, but they will also evolve over time as PHRs evolve and grow more sophisticated.

The exact shape of future information technology applications is impossible to predict. Nonetheless, the PHR appears to be certain to have a key place at the table, since it will allow individuals to increase the quality of their lives by managing their own health information, a central point on which our participants agreed. The accurate understanding and categorical analysis of opinions of those experts who lead the development and growth of PHR presented in this study should inform the adoption and expansion of the PHR, thus ensuring its widespread uptake and clinical success.

## Multimedia Appendix 1

PHR Q-samples.

## Abbreviations

EHR: electronic health record
EMR: lectronic medical record
PHR: personal health record
