# Supplementary material for: Analysis of the Definition and Utility of Personal Health Records Using Q Methodology
Source: J Med Internet Res. 2011 Nov 29;13(4):e105. doi: 10.2196/jmir.1781 (PMC3278091; doi:10.2196/jmir.1781)
Supplement: Supplementary file 1 [file jmir_v13i4e105_app1.pdf]

	Q-samples for PHR concepts in English
1	It allows various medical institutions to exchange information
2	It provides the hospital and pharamcy information
3	it provides my medical record information
4	It provides personalized health information
5	It allows me to apply for medical counseling
6	It allows me to make appointments to the hospitals
7	It has the effect of reducing health cost in a national scale
8	It realizes personalized medicine
9	It realizes participatory medicine
10	It considers the convenience from the user perspective
11	It is information for u-health services
12	It will be costly to manage
13	It is a practical business model to charge a monthly fee to the user
14	It is a practical business model to manage a sponsorship through medical institution, insurance companies, or corporations
15	It is a pratical business model to manage it through advertising revenue
16	It is a practical business model to charge insurance companies, pharmaceutical companies, and medical research facilities for the exchange of information
17	A critical mass of customer base is cruicial for the sustainability of business
18	Compensations for PHR management needs to be made through the medical insurance
19	It is possible to developt a successful business model by implementing PHR service for smartphones
20	It is comprehensive and integrative
21	It is the entire medical record of a person
22	Each individual manages their own health records
23	Legal representatives appointed by an individual can manage their health records
24	It is not a legal document
25	It legally belongs to each individual
26	It is possible to recognize who entered, accessed and edited the information
27	It is a shift in healthcare paradigm
28	It is a practical realization of future medicine
29	It is Health 2.0 where the health consumers participate
30	It is the customer-centered
31	It needs to be portable
32	It must be inter-connected
33	It must be protected by personal privacy act
34	It must be accessible at any time and from any where, when needed
35	Standardization is crucial
36	Securing privacy issue is cruicial
37	The credibility of information is crucial
38	The completeness of information is crucial
39	Certification issued by government is crucial
40	There exists a type where it is provided by medical institutions
41	There exists a type where it is provided by health insurance companies
42	There exists a type where it is provided by employers
43	There exists a type where it is provided by corporations such as Microsoft or Google
44	It is paper based
45	It is computer based
46	It is internet based
47	It is portable storage device based
48	It is most effective when it is managed by the hospital website
49	It is most effective when managed in parallel with home healthcare
50	It is most effective to port it in a USB memory device
